# A spatiotemporal analysis of the spread of African swine fever in Vietnam in 2019

**DOI:** 10.3389/fvets.2026.1858865

**Published:** 2026-07-08

**Authors:** Vo Dinh Chuong, Rachel A. Schambow, Chia-Hui Hsu, Nguyen Thi Diep, Phan Quang Minh, Hoang Manh Tien, John M. Humphreys, Andres M. Perez

**Affiliations:** 1 Vietnam Department of Animal Health and Production, Hanoi, Vietnam; 2 Center for Animal Health and Food Safety, College of Veterinary Medicine, University of Minnesota, Saint Paul, MN, United States; 3 US Department of Agriculture, Agricultural Research Service, National Bio and Agro-Defense Facility, Manhattan, KS, United States

**Keywords:** African swine fever (ASF), disease spread, epidemiology, spatiotemporal cluster analysis, Vietnam

## Abstract

**Introduction:**

African swine fever (ASF) was first detected in Vietnam in early February 2019 and spread rapidly nationwide. By December 2019, ASF had been reported in 8,509 of 11,055 communes (77%) across all 63 provinces and municipalities.

**Methods:**

Descriptive analyses were performed to summarize temporal and spatial patterns of ASF-affected communes (outbreaks). Outbreak data were aggregated weekly (week 1–48) based on onset date, and weekly counts, cumulative numbers, and proportions of affected communes were calculated. To quantify the early spread of ASF, we analyzed commune-level outbreak data from 2019. Temporal progression was assessed using linear and negative binomial regression models, whereas spatiotemporal clusters were identified using a space–time permutation model.

**Results:**

The linear regression analysis indicated an approximately 2.5 percentage-point weekly increase in the cumulative proportion of affected communes (*R*^2^ = 0.94) from 1 February to 14 July 2019. The negative binomial regression model estimated an approximately 7.6% weekly increase in affected commune counts (*β* = 0.073) from 1 February to 14 July 2019, corresponding to a doubling time of approximately 9.5 weeks. Consequently, more than half of the communes were affected within the first 24 weeks of the first detected case. A total of 18 statistically significant spatiotemporal clusters were identified. The earliest cluster was detected in the North, where the first outbreak was confirmed, and the most numerous clusters were in the Central region. Many cluster centers were located in areas of relatively high pig density, particularly in the Red River Delta and Mekong River Delta.

**Conclusion:**

These findings highlight the extensive geographic scale of ASF transmission in Vietnam in the first year of the epidemic. The rapid and extensive spread observed suggests that a stamping-out strategy, as typically implemented, may face substantial challenges under such epidemic conditions. Instead, the results emphasize the importance of impact-mitigation strategies and long-term management approaches, including enhanced biosecurity, farmer support, and measures to maintain a stable pork supply. These findings also provide relevant insights for countries facing similar endemic conditions or at risk of ASF introduction.

## 1 Introduction

African swine fever (ASF) is currently one of the most significant threats to the swine industry worldwide, particularly in Southeast Asia. ASF is notifiable to the World Organisation for Animal Health (WOAH, founded as the OIE). ASF has caused a substantial economic impact due to disruptions to pig production and international trade, as its presence results in immediate suspensions or restrictions on the trade in pigs and pork (1, 2). ASF is caused by the ASF virus (ASFV), a large, enveloped, double-stranded DNA arbovirus (3–5). ASFV is the only member of the *Asfarviridae* family, *Asfivirus* genus. The virus has been classified into 24 distinct genotypes, most of which are endemic to Africa (6), whereas only genotypes I and II have caused major outbreaks outside the continent (7–9). Members of the *Suidae* family, including domestic and wild pigs, are susceptible to ASFV. Natural transmission cycles of ASFV involve domestic pigs, wild boars, wild African suids, and soft ticks (2, 10). ASFV transmission has been associated with human-mediated activities, particularly the movement of infected pigs and swill feeding of contaminated pork products, as the virus can survive for long periods in pork products (11). ASFV does not infect humans and is not considered a food safety risk (12, 13); however, the disease poses substantial threats to food security.

ASF was first described in Kenya in 1921. Traditionally confined to the African continent with only sporadic incursions outside the continent, since 2005, ASF has been reported in more than 80 countries in Africa, Asia, Europe, North America, and the Caribbean (12). In August 2018, ASF was detected in China, which represented the first occurrence of ASF in Asia (12, 14, 15). Vietnam confirmed its first outbreak on 1 February 2019 in Hung Yen province in the Northern region, and in less than 1 year, the disease had affected 8,509 of 11,055 communes across all 63 provinces and centrally governed municipalities. The epidemic resulted in the death or culling of nearly six million pigs, representing approximately 9% of the national pig population during the first 11 months of the epidemic (16–18). More than 90% of the outbreaks occurred on backyard and small-scale farms, whereas commercial farms with stronger biosecurity measures have been less affected (16, 19).

Vietnam is one of the largest pig-producing countries in Southeast Asia. Before the introduction of ASF, the national pig herd exceeded 31 million heads in December 2018, and declined to 24.6 million by April 2020 following the epidemic. In 2018, the pig production sector in Vietnam was dominated by small-scale farms, with approximately 2.5 million households accounting for 65% of the national herd (17). Pig density was and remains highest in the Red River Delta (in the North) and the Mekong River Delta (in the South), the two most agriculturally and demographically intensive regions of the country (Supplementary Figure S1).

The Vietnamese animal health system operated within a hierarchical administrative structure, which directly influenced the organization of animal disease reporting. At the time of ASF introduction, the Department of Animal Health (DAH; currently the Department of Animal Health and Production) under the Ministry of Agriculture and Rural Development (MARD; recently renamed the Ministry of Agriculture and Environment) was responsible for coordinating national disease control policies. At the regional level, seven Regional Animal Health Offices (RAHOs) oversaw disease management within their respective jurisdictions. At the provincial level, each province maintained a Sub-Department of Animal Health (SDAH), whereas more than 700 District Veterinary Stations (DVS) operated at the district level. At the local level, each commune typically had a veterinary team, including animal health workers (AHWs) based in individual villages (20, 21). The district-level veterinary stations were dissolved after July 2025.

Animal disease reporting in Vietnam is governed by the Veterinary Law and Circular No. 07/2016/TT-BNNPTNT dated 31 May 2016, issued by the Ministry of Agriculture and Rural Development (MARD), which regulates the prevention and control of terrestrial animal diseases. The legal documents define notifiable diseases, reporting flows, timelines, and administrative responsibilities (22, 23). Outbreak reporting primarily relies on a routine surveillance system that begins at the village level. When farmers observe sick or dead pigs, they report the event to a village-based AHW, who is part of the commune veterinary team. A commune veterinarian then visits the farm to assess the situation. If the case is suspected to be a notifiable disease, it is reported to the DVS, which then initiates an official outbreak investigation. A district veterinarian conducts field verification, collects samples, submits them to an appropriate laboratory for diagnosis, and reports findings to higher administrative levels. Through this process, animal health workers play a central role in both passive and active disease surveillance systems (20). Prior to 2021, implementation of this regulatory framework relied primarily on paper-based reporting from the commune to district, provincial, and national levels. This hierarchical structure required sequential administrative transmission and manual data compilation.

Despite the implementation of extensive control measures, ASF continues to circulate nationwide (24), with potential regional heterogeneity in outbreak frequency, timing, and severity. Such variation suggests that the persistence and propagation of ASF might be influenced by differences in farm management practices, pig population densities, and the consistency of regulatory enforcement across regions. The first year of the ASF epidemic represents a critical period for understanding disease dynamics, as widespread transmission occurred alongside the persistence of a substantial proportion of uninfected communes. Analysis was restricted to 2019 to characterize the initial invasion phase, before large-scale depopulation and policy changes could modify transmission patterns. This paper aims to characterize the spatial and temporal patterns of ASF spread during that initial phase of the epidemic. Understanding the early epidemiological dynamics of ASF provides evidence to identify and prioritize resources in high-risk areas (hotspots) to mitigate disease impacts.

## 2 Materials and methods

### 2.1 Case definition and data sources

According to WOAH, an outbreak is defined as “the occurrence of one or more cases in an epidemiological unit.” An epidemiological unit refers to a group of animals with a similar likelihood of exposure to a pathogenic agent (1). These animals may share the same environment or be under a common management practice, making it likely that a pathogen in one group would quickly spread to others. While an epidemiological unit is typically a herd, it may also encompass broader groupings, such as animals owned by households within a village (25).

Following those definitions and given the high proportion of small-farm swine production in Vietnam, communes are used as the epidemiological unit for ASF outbreaks. Vietnam is divided into three major regions, referred to as North, Central, and South Vietnam, respectively (Supplementary Figure S2). Each region contains provinces and municipalities. Districts are the second level under provinces, and communes are the smallest administrative level. Communes, wards, and towns are all classified as commune-level administrative units (Supplementary Figure S3). The number of communes in each province and the administrative boundary shapefiles for Vietnam were obtained from the Statistical Yearbook of Vietnam (26) and the Global Administrative Areas (GADM) database (27), while commune sizes were derived from the shapefiles. As of 2019, the country had 63 provinces and municipalities, and 11,055 communes (26), with a median commune area of 14.5 square kilometers (km^2^) [interquartile range (IQR): 6.1–35.8 km^2^]. The distribution of communes is uneven: the North accounts for 45% of all communes, the Central region for 32%, and the South for 23%. Although communes were treated as homogeneous epidemiological units, we acknowledge that substantial within-commune variation may exist. Because communes vary markedly in geographic area, original commune polygons were retained for mapping and visualization, while geographic centroids were used to represent communes in the SaTScan space–time analysis. This approach was consistent with the commune-level resolution of the outbreak data. Therefore, detected space–time clusters were interpreted as clusters of affected commune-level units rather than precise locations of infected farms or herds within communes.

In Vietnam, a commune-level ASF outbreak was defined by DAH as the presence of at least one pig exhibiting clinical signs consistent with ASF, with laboratory confirmation of ASFV by PCR or Real-time PCR. If multiple outbreaks occurred within the same commune, that commune was considered a single ASF event. Subsequent herds within the same commune that showed typical clinical signs were considered ASFV-infected and culled without laboratory confirmation. This operational definition has been applied in previous studies of animal diseases in Vietnam, including avian influenza (28, 29).

The Vietnam government’s Department of Animal Health and Production (DAHP) ASF outbreak data used for this analysis included information at commune spatial resolution on 8,509 affected and 2,546 unaffected communes (a total of 11,055), and the observation period was from 1 February 2019 to 31 December 2019. The recorded date of an outbreak corresponded to the earliest date on which pigs exhibited clinical signs. The data had been processed by the Sub-Department of Animal Health and Production for each of the 63 provinces and then combined into a single database by the Epidemiology Division of DAHP in Hanoi, Vietnam. Commune-level swine population data were not available from the government or the national statistical resources for the study period.

### 2.2 Descriptive data analysis

Outbreak data were aggregated by week based on the recorded onset date (the earliest date when clinical signs were observed). Weekly counts of newly affected communes were calculated, along with cumulative numbers and proportions of affected communes over time. Commune-level outbreak events were aggregated into 7-day time intervals to reduce potential weekday-related reporting artifacts (Monday effects) (30). The monthly outbreak proportion was also computed to compare with the weekly proportion. The cumulative proportion of affected communes was calculated by dividing the cumulative number of affected communes by the total number of communes nationwide. A choropleth map was created in ArcGIS Pro to show the communes with ASF outbreaks.

### 2.3 Temporal analysis

To characterize the early temporal dynamics of ASF spread in Vietnam, analyses were restricted to the first 24 weeks (from 1 February to 15 July 2019) following the initial outbreak detection. This period represents the early epidemic expansion phase, during which the cumulative proportion of affected communes increased rapidly and approximately linearly. After week 24 (5.5 months), more than 50% of Vietnam’s communes were affected by ASF. From week 25, the epidemic trajectory began to plateau and fluctuate. Restricting the analysis to weeks 1–24 yielded a more accurate estimate of early epidemic growth dynamics and met the 50% cut-off for total communes affected. After week 24, the cumulative proportion exceeded 50%, and the weekly incidence began to plateau; the exponential growth assumption would thereafter be violated.

The relationship between time (cumulative week, independent/explanatory variable) and the cumulative proportion of affected communes (dependent/response variable) was assessed by fitting a simple linear regression model. The regression coefficient (slope) quantified the weekly rate of spread during the linear increase in newly infected communes, and 95% confidence intervals were obtained from the fitted model. However, because of autocorrelation, linear regression was used only descriptively to estimate the slope, and no inference was attempted.

Weekly counts of affected communes from week 1 to week 24 were also analyzed using negative binomial regression model. The variance of weekly outbreaks exceeded the mean, indicating overdispersion, a condition the model assumes (31, 32). The exponentiated regression coefficient was interpreted as the weekly relative increase in outbreaks, and the epidemic doubling time was calculated using the formula:


ln(2)/β

Where *β* is the regression coefficient (slope) of the negative binomial regression model.

### 2.4 Spatiotemporal analysis

Spatiotemporal clustering of ASF-affected communes was analyzed using a space–time permutation model. This methodology was used because only reported cases were available, while data on the population-at-risk of ASF were either unavailable or unreliable (33). The space–time scan statistic uses a cylindrical scanning window, with the circular base representing geographic area and the height representing time. The window is systematically moved across outbreak locations to identify areas and periods where observed cases exceed the number expected under random spatial and temporal distribution.

For outbreak data, each commune was represented by its geographical centroid, while commune centroids derived from the administrative boundary data were used as spatial coordinates in the model. We selected a 50% spatial window, the standard upper bound recommended in SaTScan (30, 34), to avoid missing large clusters while still allowing the algorithm flexibility to detect smaller ones. The 3-month temporal window was chosen to align with the observed epidemic dynamics of ASF in Vietnam, where local transmission and reporting typically occurred over several weeks to a few months. This choice balanced capturing meaningful transmission waves while preventing the merging of distinct outbreak periods.

The log-likelihood ratio statistic was used to assess whether a scanning window represented a significant cluster. Statistical significance of detected clusters was evaluated using 999 Monte Carlo simulations at a significance level of 0.05. The observed-to-expected ratio was calculated as the ratio of observed to expected ASF outbreaks within a cluster, representing the level of infection risk relative to areas outside the cluster.

### 2.5 Sensitivity analyses and model diagnostics

Sensitivity analyses were conducted for both temporal and spatiotemporal analyses to assess the robustness of the findings. For the temporal analysis, linear and negative binomial regression models were evaluated across time windows (Week 1–22, Week 1–24, Week 1–31, and Week 6–22) to capture distinct phases of the epidemic. These intervals were selected based on the observed epidemic curve and to evaluate the consistency of model estimates under varying temporal scopes. For the spatiotemporal analysis, the space–time permutation model was assessed using a range of spatial and temporal window sizes under different parameter settings in SaTScan.

Model diagnostics were conducted through visual inspection of residual plots and formal statistical tests for normality, heteroscedasticity, and autocorrelation.

### 2.6 Software environment

Commune-level outbreak data were processed and managed using Microsoft Excel. Statistical analyses were conducted in R version 4.3.3 using RStudio version 2023.12.1.402. The space–time permutation model was implemented in SaTScan version 10.3.2. The locations and spatial extents of statistically significant clusters were visualized using ArcGIS Pro version 3.2.0 (ESRI Inc., Redlands, CA, United States).

## 3 Results

### 3.1 Spatial distribution of ASF outbreaks

**Figure 1:**
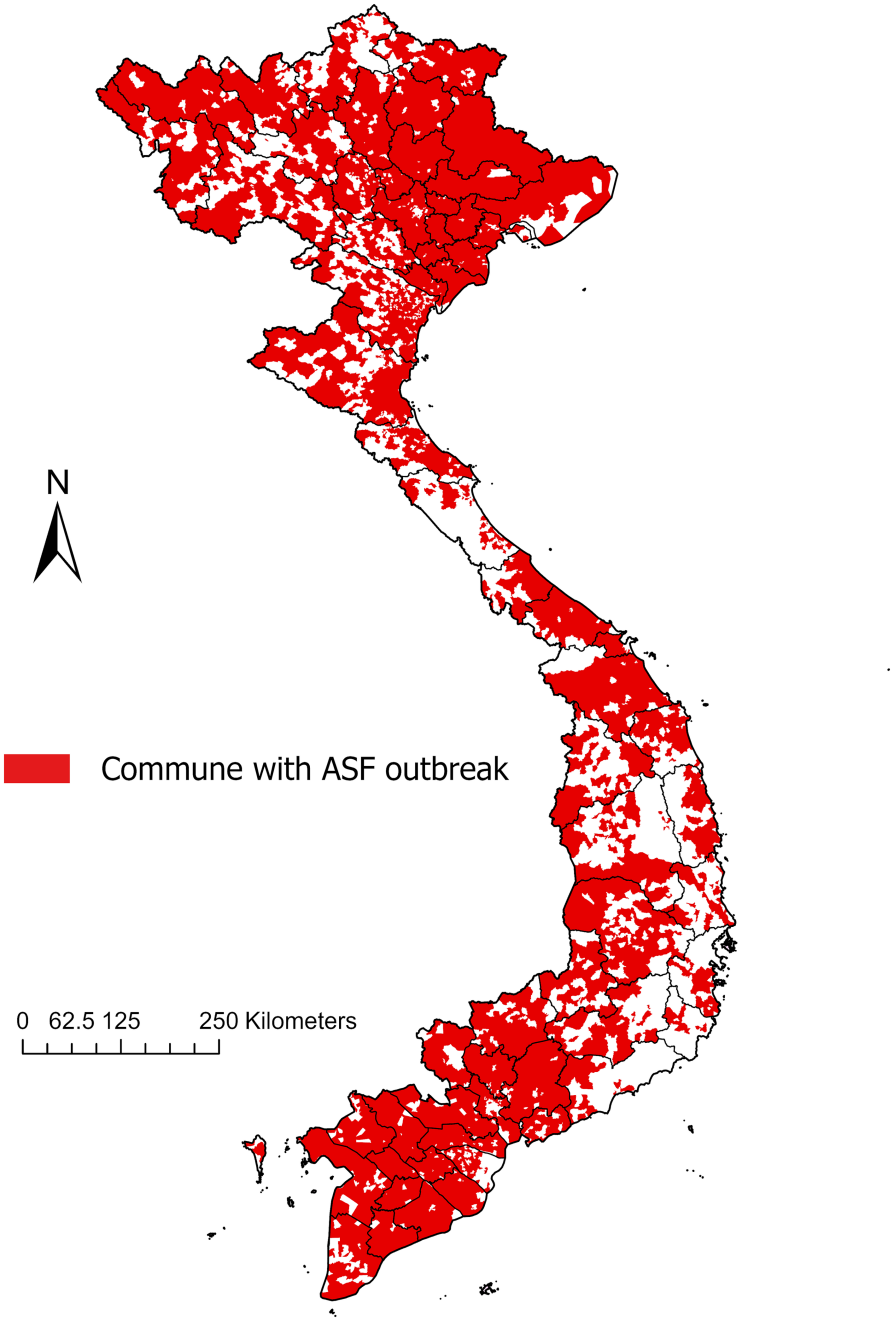
Spatial distribution of commune-level ASF outbreaks in Vietnam, 2019.

From 1 February to 31 December 2019, a total of 8,509 of 11,055 communes (77%) reported ASF outbreaks. Outbreaks were distributed across all 63 provinces and municipalities of the three major regions, including 25 in the North and 19 each in the Central and the South. The proportion of affected communes was highest in the North (85%), followed by the South (78%) and the Central region (65%). The North also accounted for the largest share of the national total (50%) due to its larger number of communes (Figure 1; Table 1).

**Table 1:** Distribution of ASF-affected administrative units in Vietnam, 2019.

Characteristic	Region	Total communes/provinces	Affected communes/provinces	Percentage of all affected communes/provinces (%)	Percentage of all affected communes by region (%)
All communes	National	11,055	8,509	100	77
By region	North	4,994	4,258	50	85
	Central	3,566	2,307	27	65
	South	2,495	1,944	23	78
All provinces	National	63	63	100	–
By region	North	25	25	40	–
	Central	19	19	30	–
	South	19	19	30	–

### 3.2 Temporal patterns

**Figure 2:**
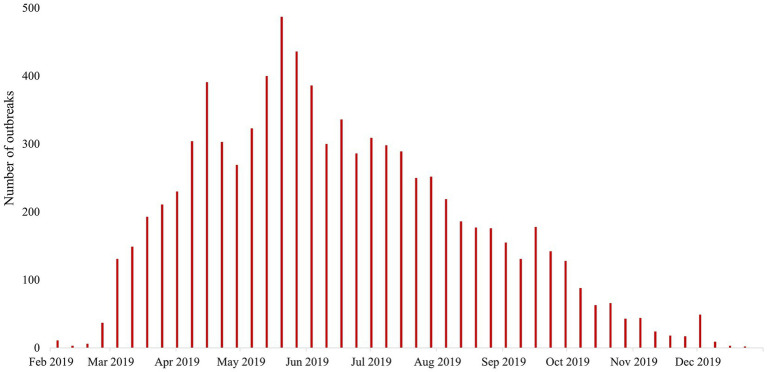
Weekly distribution of ASF outbreaks in Vietnam between 1 February and 31 December 2019.

The weekly number of outbreaks increased sharply from the beginning of the epidemic, rising from a single outbreak in the first week (February) to a peak of approximately 450–500 outbreaks per week during May–June (Figure 2). This peak was followed by a gradual decline in outbreak incidence in the subsequent months.

#### 3.2.1 Early epidemic spread

**Figure 3:**
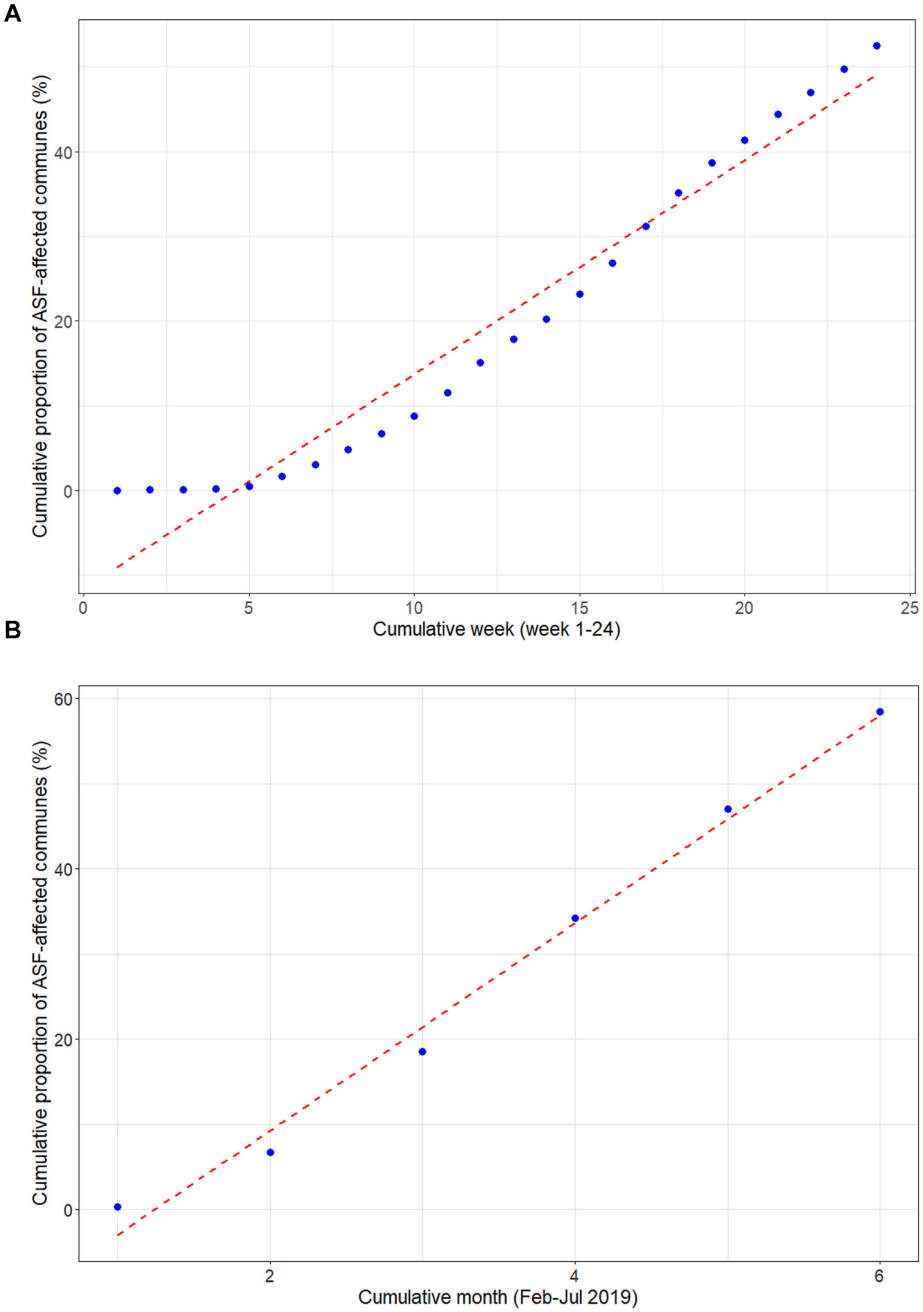
The plots show the cumulative proportion of affected communes (%) in Vietnam at monthly **(A)** and weekly **(B)** intervals from February to July 2019. The red dashed line represents the model-fitted values.

During weeks 1–24, the cumulative proportion of affected communes increased rapidly (Figure 3). After less than 6 months (the second week of July), more than 50% of communes had been affected across 61 provinces and municipalities. By 28 August 2019, all 63 provinces and municipalities in Vietnam had reported ASF outbreaks.

A linear model was used to summarize the average weekly increase in the cumulative proportion of affected communes during the first 24 weeks of the epidemic. The model estimated an average of 2.53 percentage-point per week (*β* = 2.53 ± 0.12), consistent with the monthly estimate of 12.20 percentage-point per month (*β* = 12.20 ± 0.62) (Figure 3). The adjusted *R*-squared value of 0.94 indicates that time explained a large proportion of the observed variation in cumulative affected communes over this period.

Model diagnostics indicated that some assumptions of the linear model were not fully met. Residuals did not significantly deviate from normality (Shapiro–Wilk test, W = 0.92, *p* = 0.074). However, the Breusch-Pagan test indicated significant heteroscedasticity (BP = 5.67, *p* = 0.017), and the Durbin-Watson test revealed strong positive autocorrelation (DW = 0.11, *p* < 0.001). Because cumulative epidemic data were inherently temporally dependent, the *p*-value from this linear regression was not used for formal statistical inference. Instead, the slope was interpreted descriptively as the average weekly increase in cumulative affected communes during the early epidemic period.

As a sensitivity analysis, a generalized additive model (GAM) was fitted to assess nonlinearity in the cumulative epidemic trajectory. The GAM provided a better descriptive fit than the linear model based on AIC and BIC values (AIC: 26.44 vs. 137.92; BIC: 34.65 vs. 141.46), and the smooth term indicated a nonlinear trajectory (edf = 4.94). Because residual temporal autocorrelation remained, the GAM was also interpreted descriptively as a flexible summary of the cumulative epidemic curve rather than as formal inference from independent observations.

#### 3.2.2 Weekly outbreak growth

The number of newly affected communes per week increased substantially during the early phase of the epidemic. The mean number of weekly outbreaks was 242 (median: 292; range: 1–487). The variance of weekly counts was high (21,432), indicating substantial variability in weekly outbreak counts. A negative binomial regression model was fitted to describe the association between epidemic week and the number of newly affected communes per week, allowing for potential overdispersion in the count data.

**Figure 4:**
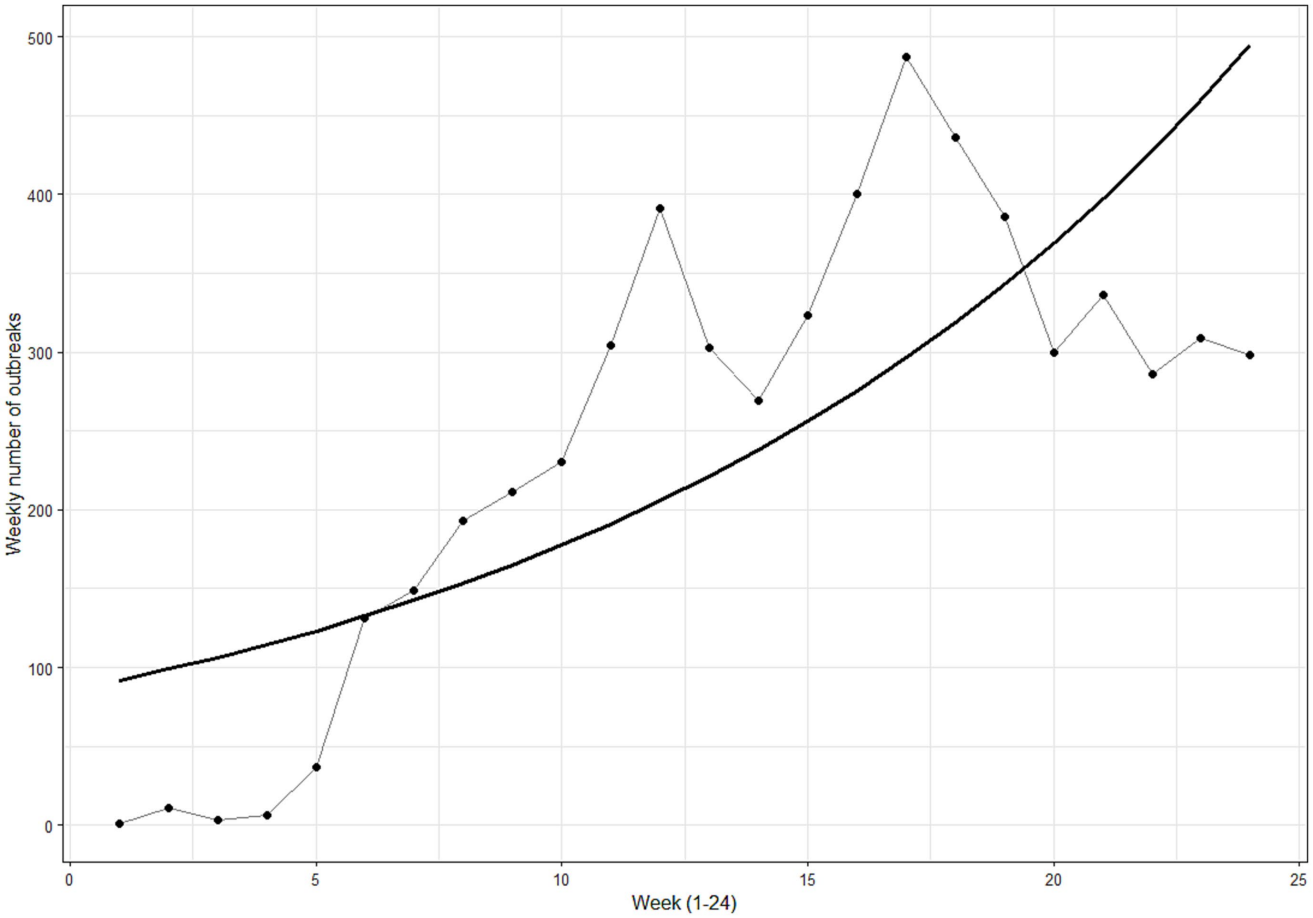
Negative binomial regression model of weekly ASF outbreak counts in Vietnam from week 1 to week 24 (February–July 2019). Points represent observed weekly outbreaks, and the solid curve represents model-fitted values, illustrating an overall exponential increase during the early phase of the epidemic.

The fitted model estimated an increase in weekly outbreak counts over time (*β* = 0.073 ± 0.002). The exponentiated coefficient (exp(*β*) = 1.076) corresponds to an average 7.6% weekly increase in outbreak counts, with an estimated doubling time of approximately 9.5 weeks. Predicted values from the model illustrate an overall exponential increase in weekly outbreaks during the initial phase of the epidemic (Figure 4).

Estimates of weekly increases were generally consistent across early epidemic time windows, including Weeks 1–22, 6–22, and 1–24. Across the windows, cumulative proportions increased by approximately 2.5–3.0 percentage-point per week, while weekly outbreak counts increased by 5.5–7.6% (Table 2).

**Table 2:** Sensitivity analysis comparing linear regression (LM) and negative binomial (NB) model estimates of weekly changes in the cumulative proportion of ASF-affected communes and weekly outbreak counts across different time windows in Vietnam, 2019.

Time period (weeks)	Weekly increase in cumulative proportion (percentage-point) - LM	Incidence rate ratio (IRR) - NB	Weekly percentage change in outbreaks (%) - NB
1–22	2.59	1.056	5.55
1–31	2.44	1.096	9.55
6–22	3.00	1.055	5.50
1–24	2.53	1.076	7.60

Residual autocorrelation diagnostics indicated significant short-lag temporal autocorrelation, with Ljung-Box tests significant at lag 1 (*χ*^2^ = 17.53, *p* < 0.001), lag 2 (*χ*^2^ = 26.55, *p* < 0.001), and lag 4 weeks (*χ*^2^ = 30.95, *p* < 0.001). These findings indicate that the independence assumption among weekly counts was not fully satisfied. Therefore, the weekly growth estimate was interpreted as a descriptive summary of early epidemic growth rather than as formal inference from a fully specified time-series model. The autocorrelation function (ACF) of Pearson residuals from the negative binomial regression model fitted to weekly outbreak counts is shown in Supplementary Figure S4.

### 3.3 Spatiotemporal patterns

**Figure 5:**
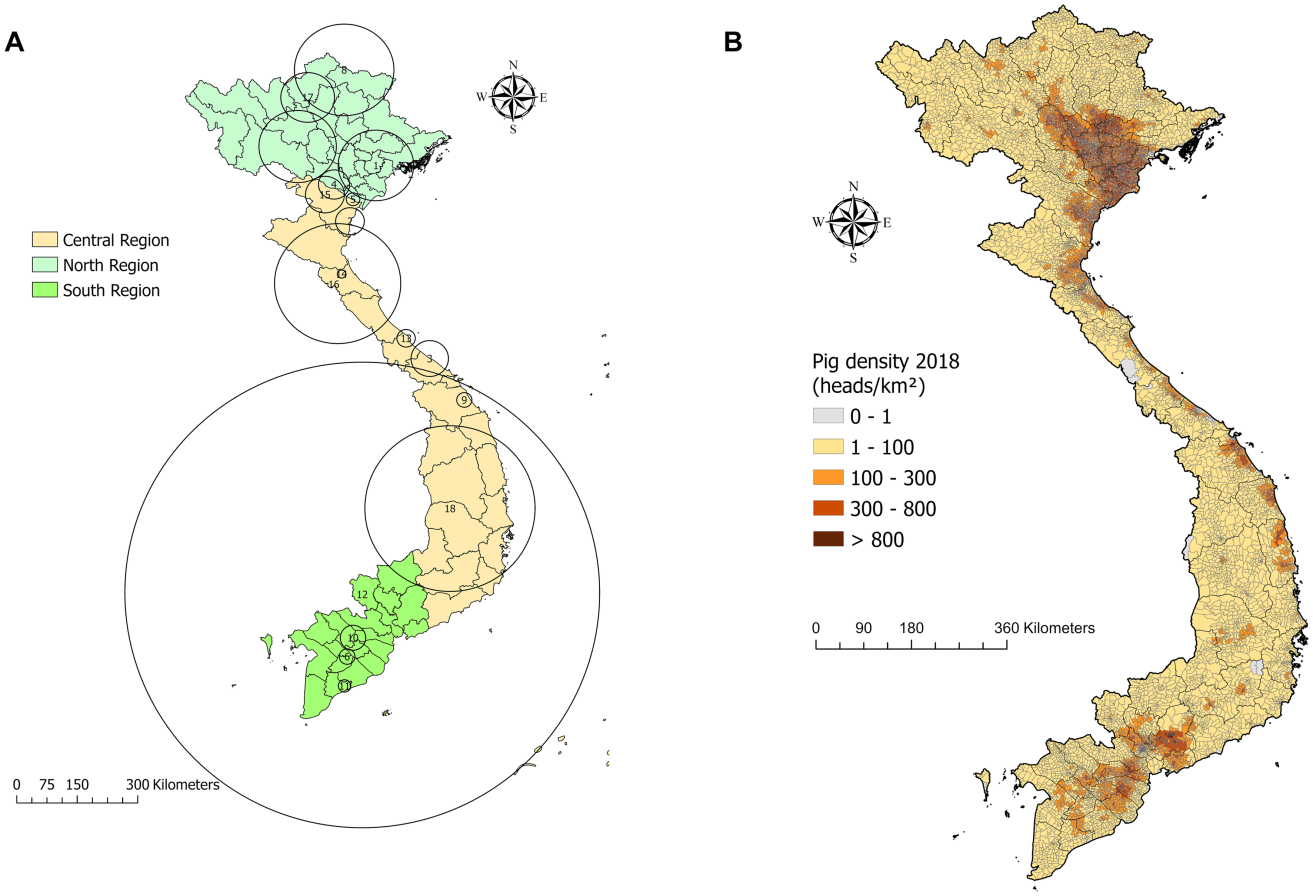
**(A)** Distribution of significant spatiotemporal clusters (*p* < 0.05) of ASF outbreaks in Vietnam from February 1 to December 31, 2019. Circle size represents the spatial extent (radius) of each cluster, and the number of clusters indicates the chronological order of detection. **(B)** Pig density at the commune level (number of pigs per square kilometer).

A total of 18 significant spatiotemporal clusters of ASF outbreaks were identified across Vietnam in 2019 (*p* < 0.05) (Table 3; Figure 5). The first cluster (Cluster 1) occurred in the North region between 13 February and 7 May 2019, covering Hung Yen and Thai Binh provinces, where the earliest outbreaks were reported. This cluster included 1,861 affected communes and had an observed-to-expected (O/E) ratio of 3.19.

**Table 3:** Significant spatiotemporal clusters of ASF outbreaks in Vietnam between February 2019 and December 2019.

Cluster ID^*^	Radius (km)	Region	Timeframe	Number of ASF-affected communes	O/E ratio	*p*-value
1	87.30	North	13 February to 7 May 2019	1861	3.19	<0.001
2	33.52	Central	8 May to 28 May 2019	114	3.56	<0.001
3	44.35	Central	15 May to 11 June 2019	99	3.95	<0.001
4	36.12	North	15 May to 25 June 2019	82	2.47	<0.001
5	15.82	North	15 May to 4 June 2019	34	3.49	<0.001
6	19.12	South	22 May to 11 June 2019	50	4.84	<0.001
7	89.24	North	29 May to 2 July 2019	275	2.18	<0.001
8	113.29	North	29 May to 18 June 2019	115	2.33	<0.001
9	18.11	Central	29 May to 11 June 2019	31	6.28	<0.001
10	30.98	South	29 May to 11 June 2019	47	2.95	<0.001
11	15.23	South	29 May to 11 June 2019	16	6.60	0.0045^**^
12	576.03	South	19 June to 10 September 2019	2005	2.00	<0.001
13	21.70	Central	17 July to 13 August 2019	25	4.93	<0.001
14	10.03	Central	21 August to 3 September 2019	15	14.32	<0.001
15	45.21	Central	4 September to 26 November 2019	51	3.19	<0.001
16	148.32	Central	11 September to 3 December 2019	291	4.62	<0.001
17	61.48	North	11 September to 26 November 2019	57	3.17	<0.001
18	204.99	Central	2 October to 24 December 2019	146	3.72	<0.001

^*^The cluster numbers were depicted chronologically.

^**^This cluster contained only 16 affected communes, which may explain its slightly lower significance.

The mean cluster radius was 67.2 km in the North, 65.8 km in the Central region, and 160.3 km in the South. The median radius in the South (25.1 km) was substantially lower than in the North (74.4 km) and Central region (38.9 km), indicating a skewed distribution influenced by a single large cluster.

ASF outbreaks were initially concentrated in the North and subsequently expanded nationwide, with substantial temporal overlap of clusters across regions during the epidemic peak. Multiple clusters were detected across all regions, with a marked concentration between May and June 2019. During this period, clusters were observed concurrently in the North, Central, and South, indicating widespread geographic distribution of outbreaks. Many cluster centers were located in areas of relatively higher pig density, particularly in the Red River Delta and Mekong River Delta (Figure 5).

The Central region accounted for the largest number of clusters (8 of 18), which occurred over an extended period from May to December 2019. These clusters were generally smaller in spatial extent but exhibited higher O/E ratios compared to clusters in other regions. Four clusters were identified in the South region, primarily between late May and June 2019. Among these, Cluster 12 was notable for its large spatial extent and long duration (19 June to 10 September 2019). The cluster spans over a radius of 576.03 km and covers 2,005 affected communes with a relatively lower O/E ratio (2.00).

Overall, most clusters were concentrated during the mid-epidemic phase (May–June 2019), followed by fewer but more spatially extensive clusters in later months.

We performed a sensitivity analysis by setting the spatial window to radii of 50 km and 100 km, with a temporal window of 60 days, to account for the potentially observed “edge effect” in the primary settings, consistent with Vietnam’s elongated shape. The number of significant clusters detected increased to 58 and 41, respectively, with a distribution pattern similar to that of the clusters found in the initial setting, but their sizes were much smaller.

## 4 Discussion

The present study, for the first time, provides a comprehensive assessment of the epidemiological patterns of ASF spread in Vietnam during the 2019 epidemic using the official national surveillance data. By integrating descriptive, temporal, and spatial analyses, the study highlights the rapid nationwide dissemination of ASF and identifies patterns in outbreak progression across regions.

At the time of ASF introduction, Vietnam comprised 11,055 communes with substantial variability in size. Pig production was predominantly based on small-scale and backyard systems, particularly in the Red River Delta, which accounts for approximately 28% of the national pig population (35). The high density of smallholder farms, combined with informal production practices and limited biosecurity, likely facilitated rapid transmission. Given these characteristics, commune-level reporting represented the most appropriate and operationally feasible epidemiological unit.

ASF spread across all 63 provinces within a short period, highlighting the intensity of transmission during the early epidemic phase. The higher burden observed in the North is likely attributable to early virus introduction, dense backyard farming, and high connectivity driven by frequent animal movement. Consistent with this, the most likely cluster was identified in Hung Yen and Thai Binh provinces between February and May 2019, corresponding to the location and timing of the earliest reported outbreaks. This finding aligns with previous studies reporting clusters in similar areas and a rapid increase in ASF-affected communes during the early epidemic phase (19).

Temporal analyses indicate a steep increase in outbreaks during the early phase, with the cumulative proportion of affected communes rising by approximately 2.5 percentage-point per week. By mid-July 2019, more than half of the communes had reported outbreaks, and by late August, all provinces were affected. Weekly outbreak counts increased by approximately 7.6% during the early epidemic phase, consistent with exponential growth, corresponding to a doubling time of approximately 9.5 weeks. The marked overdispersion in weekly counts suggests substantial heterogeneity in transmission dynamics, likely reflecting variation in production systems, connectivity, and biosecurity across regions. Previous studies also estimated rapid ASF transmission in Vietnam during a similar time period (*R*_o_ = 2.48–4.95) (36).

Spatiotemporal clustering analyses indicate that ASF outbreaks initially concentrated in the North before rapidly disseminating to the Central and South regions. The presence of overlapping clusters across regions during the epidemic peak suggests widespread transmission rather than localized persistence. The Central region has many smaller clusters with high O/E ratios, possibly owing to fragmented pig production or heterogeneous biosecurity leading to intense local outbreaks without extensive spatial spread. Additionally, the large spatial extent but moderate O/E ratio of Cluster 12 may indicate widespread low-level transmission across the South, in contrast to the smaller but more intense foci in the Central region. Moreover, the spatial correspondence between outbreak clusters and areas of high pig density likely supports the role of host density in facilitating transmission and outbreak amplification. This pattern is consistent with previous studies showing a north-to-south spread during the early epidemic phase, possibly driven by the movement of live pigs and human-mediated factors (16, 19).

ASF is widely considered to spread primarily through the movement of infected pigs and human-mediated activities (37, 38). In Southeast Asian countries, practices such as swill feeding, free-ranging, swine traders, poor biosecurity, inappropriate slaughter and carcass disposal, and frequent animal and human movement were identified as risk factors for ASF transmission (39–43). Some practices known as common in Vietnam may have facilitated the rapid spread of ASF, including illegal or informal transportation of pigs and pork products across borders and between regions; swill feeding; informal slaughter system; predominant small-scale farming with poor biosecurity and a high level of connectivity between farms; human and vehicle activities; and the disposal of pig carcasses in public areas. Additionally, increased pig movement and trade associated with the Tet holiday (Lunar New Year) may have further accelerated the early spread of ASF (16, 19, 35, 44–49), although direct evidence from movement data is not available in this study. These pathways could explain the rapid increase in affected communes and the early space–time clustering observed in this study. However, at the time of this manuscript preparation, very few field studies on risk factors have been conducted in Vietnam (36, 45), which revealed that the distances between affected farms and other swine farms, proximity to irrigation systems and main roads, farms located at lower elevations, poor hygienic practices by workers, and inadequate hygiene conditions at pig loading/unloading locations were risk factors for ASF outbreaks. Several statistical models suggested that higher pig density and connectivity were associated with the risk of ASF occurrence (50). The present study could not formally assess associations between these transmission-related factors and commune-level ASF occurrence because spatially explicit data on these risk factors were not available during the study period. Therefore, the detected clusters should be interpreted as space–time concentrations of affected commune-level units rather than evidence of specific transmission routes or farm-level risk factors.

The extensive geographic scale of ASF spread during the early epidemic phase may reflect both undetected circulation prior to the first reported outbreak and increased reporting following official recognition and compensation policies. The subsequent decline in reported outbreaks does not necessarily indicate disease elimination, but may instead reflect depopulation of pig herds or underreporting, particularly in areas with limited veterinary capacity or economic disincentives. Given the continued presence of pig production, recurrent ASF transmission may have occurred but was not consistently reported (45).

The scale and speed of the epidemic, combined with the economic importance of pig production, prompted a transition from eradication to mitigation strategies. While initial control efforts focused on stamping out, this approach resulted in substantial reductions in the national swine population and was difficult to sustain. Consequently, control strategies have increasingly emphasized selective culling, enhanced biosecurity, vaccination, and farmer support programs aligned with the national ASF control plan (24, 51–53).

By the end of 2019, a substantial number of communes remained unaffected. This may reflect a combination of limited pig populations (e.g., urban areas), underreporting in remote regions, and economic disincentives to report outbreaks. Geographic isolation, reduced animal movement, and improved biosecurity may also have lowered the risk of disease introduction. Further studies are needed to identify protective social, environmental, and management factors that may explain the absence of ASF in these communes (16, 45).

This study has several limitations. Firstly, the use of passive surveillance data may introduce underreporting, detection bias, and reporting inconsistencies, particularly in remote areas and during the early epidemic phase. Since communes were used as homogeneous analytical units, we acknowledge that within-commune heterogeneity (ecological fallacy) may exist. The analysis did not incorporate potential risk factors (e.g., farm density, transportation networks, biosecurity levels), which could confound the observed spatial patterns. Most outbreaks have only a first detection date, and the lack of commune-level swine population limits the choice of analytical methods and inference. There is also a possibility of misclassification of ASF and other swine endemic diseases in Vietnam, such as classical swine fever (CSF) and porcine reproductive and respiratory syndrome (PRRS). Secondly, the models also have their own drawbacks. The linear regression model was applied to cumulative proportions, which are inherently autocorrelated and only approximately linear over time. Although the negative binomial model was fitted to allow for overdispersion in weekly outbreak counts, residual temporal autocorrelation was detected, indicating that weekly observations were not fully independent. This temporal dependence is expected during an evolving epidemic, in which outbreak counts in adjacent weeks are likely correlated. Therefore, the estimated weekly growth rate should be interpreted as a descriptive summary of the early epidemic trajectory rather than as an inference from a fully specified time-series model. In addition, uncertainty in outbreak dates may introduce temporal inaccuracies, and administrative changes in 2019 may affect the accuracy of commune counts (54).

In conclusion, these findings provide important insights into ASF transmission dynamics in Vietnam and support the development of risk-based surveillance and control strategies. The results also contribute to the broader understanding of ASF epidemiology in endemic settings and may inform control efforts in countries facing similar challenges.

## Data Availability

Official data reports belong to the Vietnamese government and might be available upon request.
