# Supplementary material for: A spatiotemporal analysis of the spread of African swine fever in Vietnam in 2019
Source: Front Vet Sci. 2026 Jul 8;13:1858865. doi: 10.3389/fvets.2026.1858865 (PMC13388069; doi:10.3389/fvets.2026.1858865)
Supplement: Supplementary file 1 [file Data_Sheet_1.docx]

Supplementary Material


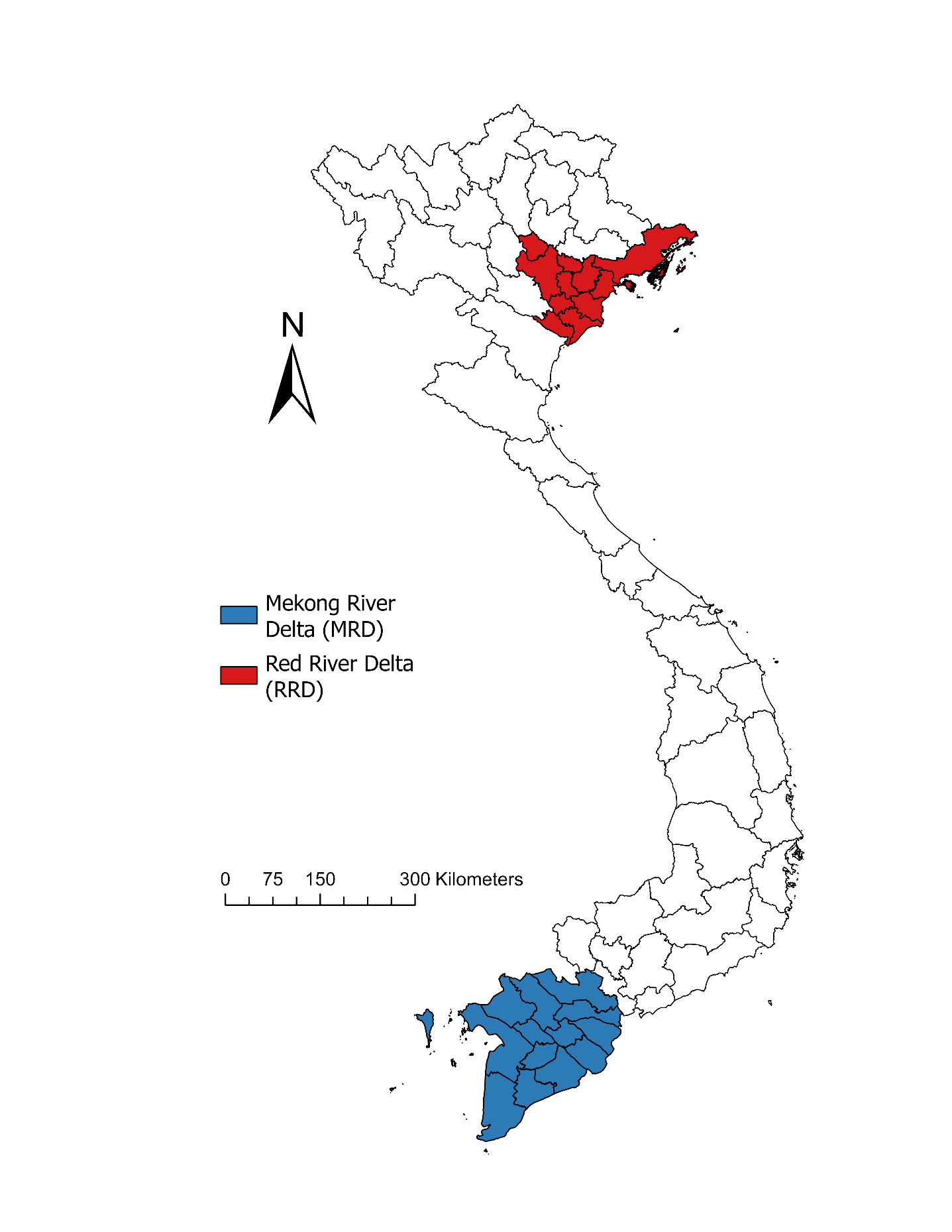


**Supplementary Figure 1.** The map shows the locations of the Red River Delta and the Mekong River Delta regions in Vietnam in 2019.





**Supplementary Figure 2.** The map shows the locations of provinces by region (North, Central, South) in Vietnam in 2019.


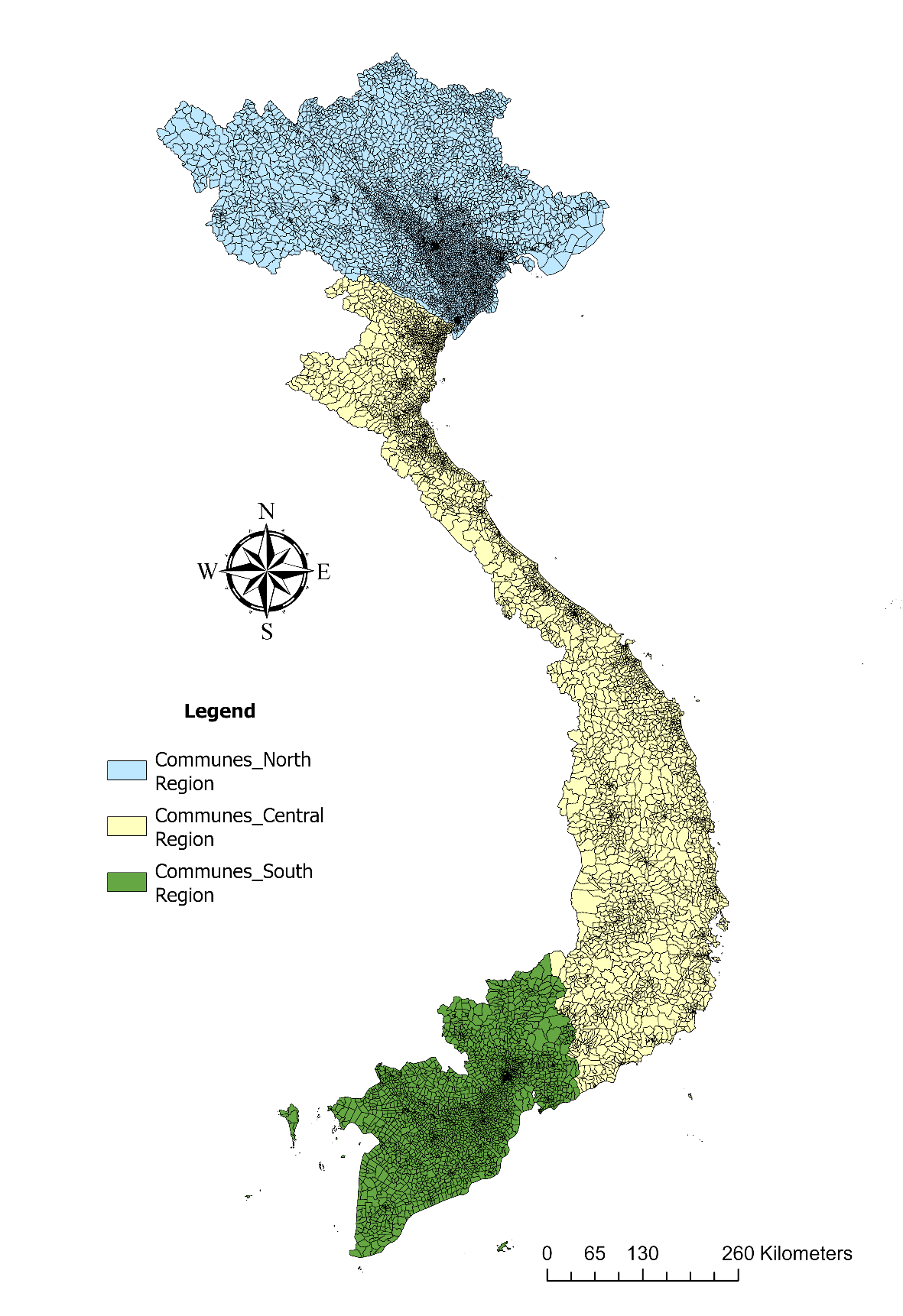


**Supplementary Figure 3.** Distribution of communes by regions (North, Central, South) in Vietnam in 2019.


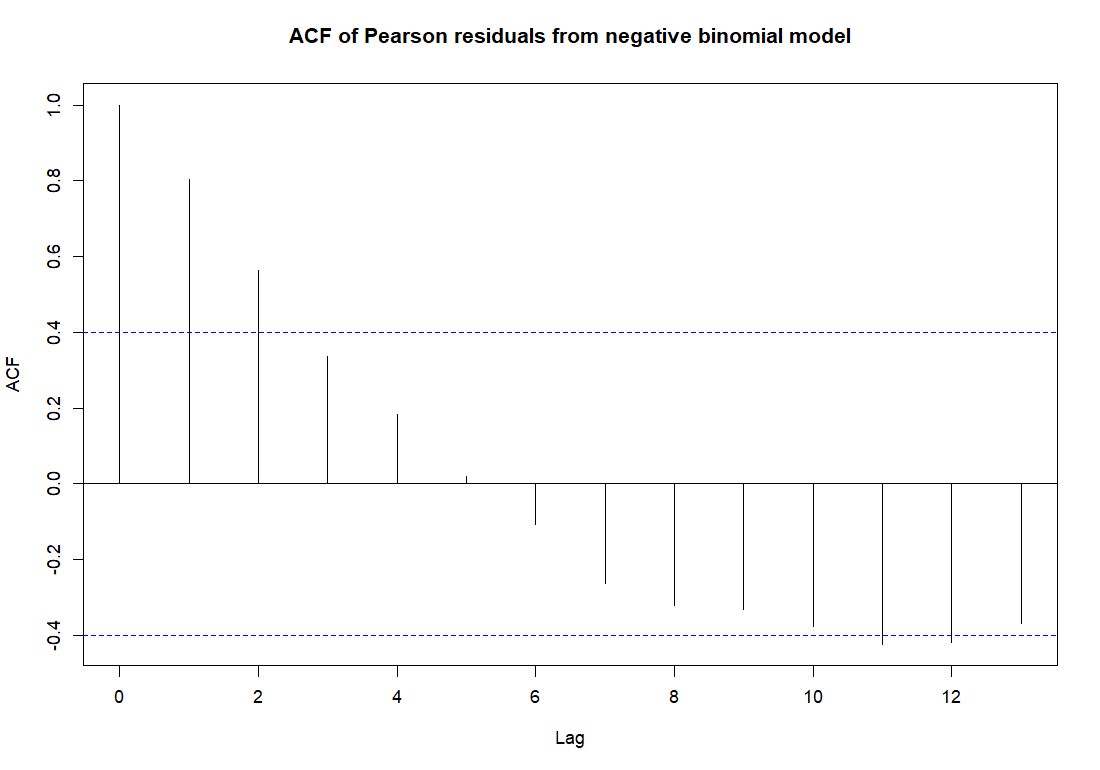


**Supplementary Figure 4.** Autocorrelation function (ACF) of Pearson residuals from the negative binomial regression model fitted to weekly outbreak counts. Bars outside the dashed confidence limits indicate residual temporal autocorrelation. The plot shows strong positive autocorrelation at short lags, indicating that the assumption of independence among weekly observations was not fully satisfied.
